# Guillain-Barré syndrome after cervical spine surgery: a case report

**DOI:** 10.1186/s40981-018-0165-2

**Published:** 2018-03-16

**Authors:** Takaaki Oku, Kengo Maekawa

**Affiliations:** Department of Anesthesiology, Kumamoto Chuo Hospital, 1-5-1 Tainoshima, Minami-ku, Kumamoto, 862-0965 Japan

## To the editor

Guillain-Barré syndrome (GBS) is an acute polyneuropathy frequently triggered by infection. It has also been reported in some case after surgical procedures. We managed a patient who developed Guillain-Barré syndrome after surgery on their cervical spine.

A 66-year-old man with bilateral numbness in his hands and gait instability underwent cervical laminoplasty (C3-6) for cervical spondylotic myelopathy. General anesthesia was induced with propofol (target-controlled infusion (TCI): 3 μg/ml), rocuronium (50 mg) and maintained with oxygen, air, propofol (TCI: 2.5–3 μg/ml), and remifentanil (0.1–0.15 μg/kg/min). Motor evoked potentials were monitored intraoperatively. No complications were apparent during the procedure. Gait training with a walker was initiated on postoperative day 3. He was able to climb stairs with a cane on postoperative day 10.

On postoperative day 13, he began to experience a slight weakness in his hands and feet. He had no constitutional symptoms of infection. Physical examination revealed areflexic paresis of all four extremities, with the symptoms worst in the proximal (manual muscle testing of 1/5) than distal portions (3/5) of his extremities. He denied any respiratory or swallowing difficulty. Magnetic resonance images of the entire spine showed normal findings. His muscle paralysis temporarily improved (2/5 strength in his upper and 3/5 lower extremities) after 1000 mg of methylprednisolone was administered for 2 days. An electrophysiological examination performed on postoperative day 24 revealed low-amplitude compound muscle action potentials with normal sensory nerve action potential. His cerebrospinal fluid had five nucleated cells per cubic millimeter and a markedly elevated level of protein 119 mg/dl (normal, 10 to 40 mg/dl). A diagnosis of acute motor axonal neuropathy (AMAN) type Guillain-Barre syndrome was established on the basis of these results. Intravenous immunoglobulin 0.4 mg/kg/day was administered for 5 days. He showed good response to treatment and his power improved, regaining 5/5 strength in his upper and lower extremities. On postoperative day 31, he was able to ambulate independently without any aids and was discharged to a rehabilitation facility.

The typical symptoms of GBS are muscle weakness in the limbs and sensory disturbances such as pain and numbness. The symptoms and signs of GBS are similar in clinical performance to those of spinal cord compression injury or nerve root compression syndrome. It has been shown that surgery can trigger GBS [1, 2]. Gensick et al. demonstrated that the relative risk of contracting GBS was 13.1 times higher in surgical patients compared to the normal population [3].

The pathophysiology of GBS in postsurgical patients is not well understood, but the common pathway involves an autoimmune insult on the nervous system. By activating the endocrine stress systems, surgery leads to transient immunosuppression, which would allow autoantibodies to promote an attack on the peripheral nerves. Exposure to certain viruses, bacteria, vaccines, or even myelin itself may sensitize the body’s immune systems, which can trigger an autoimmune response on the nervous system [4].

Making a diagnosis of GBS is difficult for most surgeons because they are not familiar with this disease. It often takes time for surgeons to recognize and confirm the condition, and the therapeutic measures specific to the condition often are delayed. In this case, GBS was diagnosed 12 days after onset. To avoid such delay, we recommend neurologic examinations when there is unexplained progressive postoperative muscle weakness. If findings in a patient with postoperative weakness cannot be explained by imaging, the differential diagnosis must be expanded to include more rare etiologies. If GBS is considered, a spinal tap showing elevated cerebrospinal fluid protein combined with nerve conduction studies that reveal slowed or blocked normal nerve conduction can confirm the diagnosis. Regular treatment of GBS to improve prognosis consists of a 5-day course of intravenous immunoglobulin at a dose of 0.4 mg/kg and/or plasma exchange. Supportive care, such as ventilator support, during and following hospitalization also is crucial.

## Abbreviations

AMAN: Acute motor axonal neuropathy
GBS: Guillain-Barré syndrome

## References

[1] Cingoz F, Tavlasoglu M, Kurkluoglu M, Sahin MA (2012). Guillain-Barre syndrome after coronary artery bypass surgery. Interact Cardiovasc Thorac Surg.

[2] Battaglia F, Sevy A, Moyse E, Roche PH (2013). Guillain-Barré syndrome following severe head trauma and spine surgery. Rev Neurol (Paris).

[3] Gensicke H, Datta AN, Dill P, Schindler C, Fischer D (2012). Increased incidence of Guillain-Barré syndrome after surgery. Eur J Neurol.

[4] Hughes RA, Rees JH (1997). Clinical and epidemiologic features of Guillain-Barré syndrome. J Infect Dis.

